# Evaluating integration in collaborative cross-disciplinary FDA new drug reviews using an input-process-output model

**DOI:** 10.1017/cts.2021.861

**Published:** 2021-09-30

**Authors:** Kevin Bugin, Gaetano R. Lotrecchiano, Michael O’Rourke, Joan Butler

**Affiliations:** 1 Office of New Drugs, Center for Drug Evaluation and Research, U.S. Food and Drug Administration, Silver Spring, MD, USA; 2 Department of Clinical Research and Leadership, George Washington University School of Medicine and Health Sciences, Washington, DC, USA; 3 Center for Interdisciplinarity, Department of Philosophy, and AgBioResearch, Michigan State University, East Lansing, MI, USA

**Keywords:** Cross-disciplinary integration, evaluation, science-of-team-science, FDA, benefit-risk assessment

## Abstract

**Background/Objectives:**

The US Food and Drug Administration (FDA) is responsible for assessing safety (risks) and effectiveness (benefits) of new drug products using the data provided in a Sponsor’s new drug product marketing application before they can be marketed. The FDA forms cross-disciplinary review teams to conduct these assessments. Recently, the FDA began implementing more interdisciplinary approaches to its assessments, reducing redundancy in review processes and documentation by increasing team integration around review issues.

**Methods:**

Through a phenomenological descriptive comparative case study, the impact of FDA’s new interdisciplinary approach on review team integration was compared with its traditional multidisciplinary review approach.

**Results:**

We identified collaborative integration occurring in one FDA review team using the new interdisciplinary review and another team using the traditional review and then modeled and analyzed the collaborative, cross-disciplinary integration in each case using an input-process-output (IPO) model drawn from the Science-of-Team-Science (SciTS).

**Conclusion:**

This study provides a systematic method for understanding and visualizing integration in each type of review previously and presently used at FDA and illustrates how the new interdisciplinary approach can ensure more integration than more traditional approaches previously used. In addition, our study suggests that an IPO model of integration can characterize how effectively FDA review teams are integrating around issues and assist in the evaluation of differences in integration between FDA’s new interdisciplinary review and the existing multidisciplinary approach. The approach used here is a new application of SciTS scholarship in a unique sector, and it also serves as an example for measuring review team effectiveness.

## Introduction

Cross-disciplinary integration is a key feature of interdisciplinary research, and the collaborative form is often a desired outcome of Team Science [1-3]. This study deployed a phenomenological descriptive comparative case study approach to identify and characterize the nature of the collaborative integration occurring in US Food and Drug Administration (FDA) review teams for two new drug product marketing applications using two different forms of cross-disciplinary research. As Julie Thompson Klein articulated in her discussion of interdisciplinarity, this integration increases across the continuum of cross-disciplinary research from unidisciplinarity to transdisciplinarity and is characterized heuristically by different forms of integration [4]. For this reason, integration is expected in a multidisciplinary case and in an interdisciplinary case, but it should be somewhat different.

The study described in this article was conducted in 2020 to comparatively evaluate integration around key review issues in the new FDA interdisciplinary review and in the traditional FDA multidisciplinary review. The goal of this study was to provide an in-depth analysis to better guide FDA’s shift to the new interdisciplinary review. Key review issues are the focus of all marketing application reviews, new and old, as they form the basis of benefit-risk assessment, that is, assessment of whether a patient taking the proposed new drug product will experience greater therapeutic benefits than risks, such as adverse reactions commonly termed “side effects.”

### FDA New Drug Product Reviews

FDA’s mission is to protect and promote US public health. One of the ways it achieves this mission is by authorizing new medical products to be marketed based on the data submitted in a marketing application to assess their safety and effectiveness [5]. In 2019, FDA began rolling out a new interdisciplinary approach to its review of marketing applications for new drugs, with the key new feature being the required use of an integrated, collaboratively written review document and a more interdisciplinary, issue-focused review team approach [6]. This approach enhances the traditional multidisciplinary review approach that involved scientists from multiple disciplines working together in a separate but coordinated fashion to assess the safety and effectiveness of a new drug product by building in earlier and more frequent collaboration and a joint review document written as a team.

Multiple FDA working groups gathered internal and external stakeholder feedback on the traditional review approach to marketing applications and identified several problems, including redundancy within review documents and an insufficient focus on the key review issues associated with marketing application reviews [7]. *Review issues* are the “problems” that the FDA review teams must work through in a cross-disciplinary way to understand and, if possible, solve [8]. These are issues related to a drug’s benefit and/or risk, and they affect approvability or lead to other FDA regulatory actions. Based on these challenges, a new more interdisciplinary approach was developed.

**Fig. 1.**
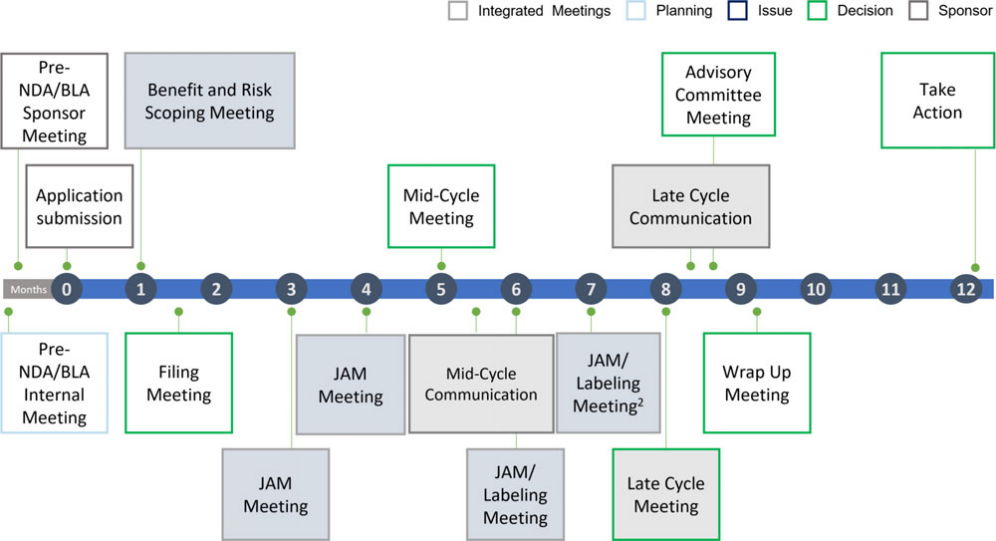
Integrated review approach: New drug application/biologic licensing application (NDA/BLA) are the focus of the review teams’ assessment. New integrated meetings are indicated: Joint assessment meetings (JAMs) are the problem-focused interdisciplinary working meetings prescribed in the new interdisciplinary review [9].

Marketing application review, both traditional and the new interdisciplinary, begins with presubmission activities, such as a pre-new drug application (NDA)/biologics license application (BLA) meeting with the new drug’s Sponsor (or manufacturer). Upon submission of the application, the review team convenes to discuss its suitability for filing (e.g., is it complete?), then typically meets again at a halfway point (i.e., the Mid-Cycle Meeting), and again late in the review cycle, and lastly to wrap up and make any decisions (i.e., Wrap Up Meeting). For some applications, an Advisory Committee Meeting is held. NDA/BLA are the regulatory terms for the marketing application for a new drug (see Fig. 1). In the interdisciplinary review, review teams conduct several new activities to promote greater integration and focus on review issues. First, a benefit-risk scoping meeting that involves the entire FDA team and senior leadership is held prior to accepting (i.e., filing) the marketing application, where review issues can be identified early and monitored for the remainder of the review. Then for each review issue identified, a joint assessment meeting (JAM) is held. JAMs are problem-focused, interdisciplinary working meetings that involve all team members with a relevant perspective on the issue; these meetings enable the team to identify and then work through the key review issues collectively, increasing collaboration and communication. Integrated JAMs are highlighted in Fig. 1.

**Fig. 2.**
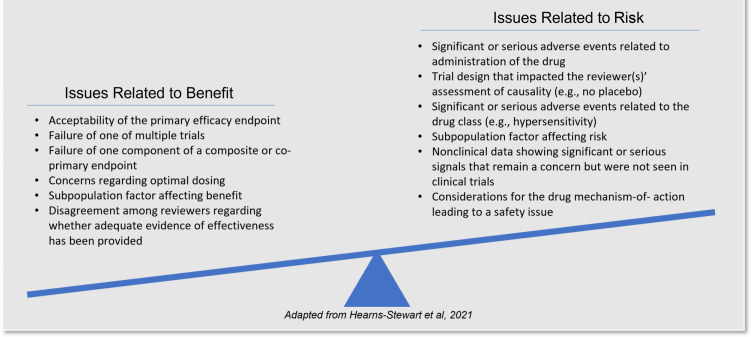
Benefit-risk assessment: Review issues are considered by individual team members and also collectively before determining that the benefits outweigh the risks, representing a favorable decision [7].

In both traditional multidisciplinary reviews and new interdisciplinary reviews, FDA’s review teams use a conceptual framework, known as the *structured benefit-risk framework* (BRF), to guide the assessment of benefit and risk for the proposed products [8]. It is through the examination of benefit-risk concepts that FDA review teams identify review issues and formulate an integrated assessment of benefits and risks, ultimately determining whether the benefits outweigh the risks in the context of the proposed treatment (see Fig. 2). The review issues encountered by teams assessing the benefits and risks of a NDA tend to concern the clinical evidence and uncertainty related to efficacy and/or safety signals, such as study endpoints, design of or results from clinical trials, dosing and pharmacokinetics, and subpopulation factors. In the new interdisciplinary reviews, review team assessments are documented in an integrated way in the new integrated review template, which replaces multiple separate, discipline-specific review templates that existed in the traditional multidisciplinary review.

FDA’s implementation of the interdisciplinary assessment of FDA new drug marketing applications has taken a phased-in approach to enable careful evaluation of its new features as its process and integrated review template are rolled out. An update on this phased approach was recently provided during a virtual public workshop in October 2020 [10], in which FDA highlighted internal and external support for the enhanced collaboration and communication that the new approach was promoting, but noted concerns with transparency in review documentation on reviewer disagreements, the independence of reviewers, and the need for more information regarding development program issues. These concerns echoed those discussed in publications earlier in the year regarding the lack of transparency and knowledge loss in the new approach [6,11]. This clearly highlights the need for a robust method of evaluating integration in the new review approach to weigh what is gained against what might be lost.

### IPO Approach to Modeling Integration

**Fig. 3.**
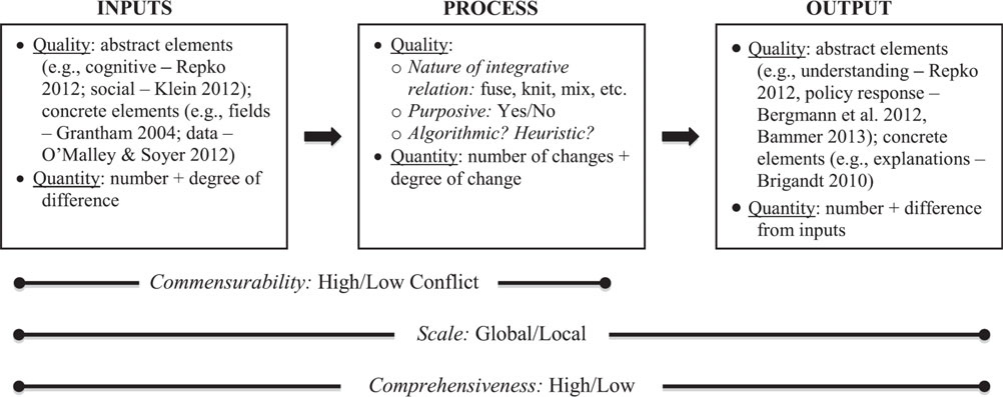
Schematic diagram of input/output process model of integration [20].

To conduct this comparative evaluation, we needed a general way of modeling integration. Although integration is widely regarded as a critical feature of interdisciplinarity [2,12,13] and has received close attention in several literatures (e.g., philosophy of science [14], biology, [15] education [16], cross-disciplinary theory [17,18], organizational psychology [19]), there are few theoretical accounts presenting a systematic framework that can be deployed to model the integration present in complex processes, such as NDA reviews. O’Rourke et al. is an exception [20]. It develops a philosophical framework and an associated input-process-output (IPO) model for cross-disciplinary integration that are grounded in a close and wide-ranging review of the literature on integration, especially in philosophy of science and interdisciplinary theory; the model is designed to synthesize insights into the nature of integration found across a large swath of this literature (see Fig. 3). Further, it is designed to be a *general* model of integration systematically applicable across a wide range of differences, such as the differences obtained between a multidisciplinary review and an interdisciplinary review [21]. Both types of review may exhibit integration, and the model used should be able to account for that [22]. Finally, the IPO character of the model in O’Rourke et al. aligns closely with the structure of NDA reviews − there are clear inputs, a review process, and judgments about the efficacy/safety of the drug as output. The model thus allows for systematic identification of integration exhibited by NDA benefit-risk reviews.

The O’Rourke et al. IPO model presents integration as a “generic combination process, the details of which are determined by the specific contexts in which particular instances of integration occur” (p. 67) [8,20]. In this “generic” process, inputs are combined in the production of outputs. This is an application of the widely used IPO model, which has proven very useful across a wide variety of domains [23,24]. The utility of the IPO model from O’Rourke et al. derives from the analytical framework it offers that can be used to organize “one’s thinking” about the inputs, outputs, and integration process variables of the model and “facilitate comparison” across these categories (p. 68) [8]. The model is sensitive to qualitative and quantitative characteristics of inputs and outputs, accommodating concrete and abstract elements and recording the number of elements and degree of difference among them. O’Rourke et al. understand integration to be a process, and so the process elements are central to the model, and in particular *integrative relations*. The integrative relations involved in the process activities, or changes to inputs, were assessed as either Synthetic or Combinatorial, relying on descriptions of these relationships from O’Rourke et al.:

**Synthetic** − brings together inputs in some way for irreversible integration. **Combinatorial** − an assembling or combining of inputs but is of low change to the inputs (e.g., stacking).

Integrative relations contrast with disintegrative relations, which reverse integration present in the inputs. (Preservative relations are third relation, but they do not figure into our analyses.) The disintegrative relation involved in the process activities is analytic:

**Analytic** – changes aimed at breaking an input down into its constituent parts or to differentiate between the inputs.

**Fig. 4.**
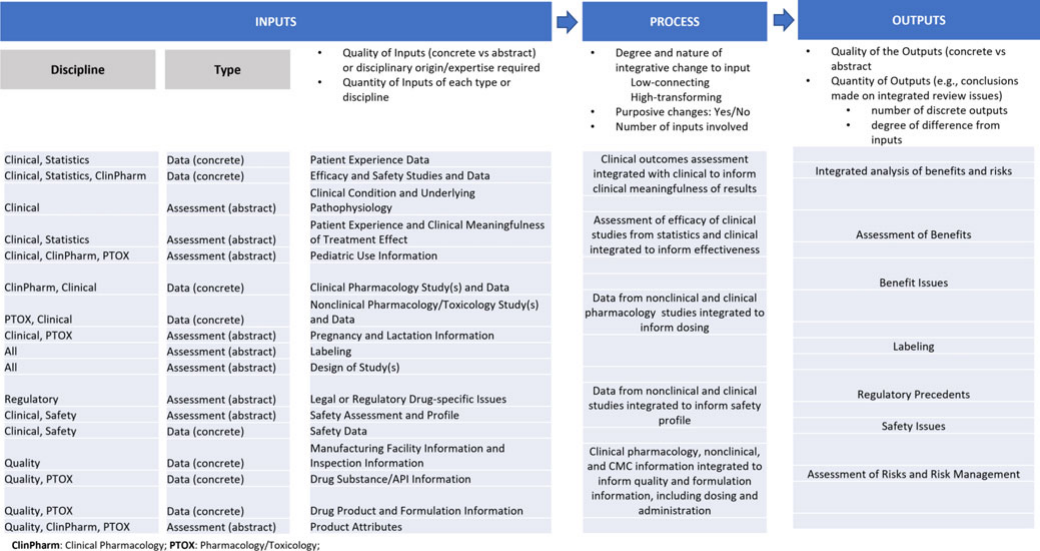
Contextualized input-process-output (IPO) benefit-risk framework: This contextualized IPO model merges the key dimensions of the benefit-risk framework (i.e., evidence and uncertainty related to review issues that influence the assessment of benefits or risks) and examples of how the inputs, process activities, and outputs would appear in the context of a new drug product marketing application assessment.

The model views integration as consisting in placing inputs into integrative relations (e.g., subsumption, serialization) in the production of outputs, where the number of inputs will typically exceed the number of outputs. The final pieces of this model are parameters (e.g., scale, comprehensiveness) that allow the model to be applied across a wide range of contexts (see Fig. 3). A contextualized IPO model, created through the coupling of the O’Rourke et al. cross-disciplinary integration IPO model with the structured BRF for new drug reviews, would further enable data collection and analysis for this study (see Fig. 4). Contextualization was possible through the FDA researcher’s experience, which was related to process knowledge of how FDA Review Teams operate, familiarity with the context of drug development, and awareness of benefit-risk review issues. This experience also helped with the coding and the analysis/assessment of different dimensions of the inputs, processes, and outputs.

## Methods

This phenomenological descriptive comparative case study targeted a completed review of one NDA using the traditional multidisciplinary approach and a completed review of another NDA using the interdisciplinary approach. The study employed a combination of document analysis, semi-structured interviews, and member checking to characterize the integration found within each case. This is a qualitative study that compares only two cases, and so some bias from case selection is possible. To minimize the potential impact of this bias, cases for evaluation in this study were selected using a sampling frame of highly similar new drug product applications, with subsequently similarly constituted teams and expected comparable levels of detail and complexity. The sampling frame was created from the reviews of new molecular entity drug product applications completed between May 2019 and May 2020. Two pools were created based on whether the team used the multidisciplinary review or the new interdisciplinary review. There were 60 applications in total in the sampling frame, with five applications receiving the new interdisciplinary review. Cases from both pools were assigned a random number, and one case from each group was selected at random using a randomizer – the RINVOQ case for the multidisciplinary review and the TAUVID case for the interdisciplinary review. This random selection of cases minimized bias even though the selection was purposive [25].

Following the identification of cases, review team members and their supervisors were contacted for recruitment purposes. Upon confirmation of participation, a case was considered “selected” and review documents were gathered from Drugs@FDA, a public database that includes FDA review documents for approved applications. Review documents were analyzed to identify benefit-risk review issues, guided by the lead researcher’s experience and the merged structured BRF and IPO model, as discussed earlier [26]. Preliminary coding was conducted to label the identified benefit-risk review issues across documents and then double-coded as associated with one of the variables of the IPO model (i.e., inputs, processes, or outputs) [27]. The initial coding of several review documents was reviewed with an interrater familiar with new drug product marketing application reviews to confirm process and initial coding.

Inputs and outputs associated with each review issue were coded as either concrete or abstract, based on document analysis. An input or output was considered *concrete* if it includes tangible, physical elements, such as data, literature, or sponsor-submitted analyses (e.g., reviewing inclusion/exclusion criteria in a protocol, or the results of prespecified statistical analyses). An input or output was considered *abstract* if it was cognitively based, such as a perspective, an expert opinion, insights, new analyses that may be needed, or conversation/discussions (e.g., clinical judgment on the clinical meaningfulness of a treatment effect or retrospective statistical analyses for sensitivity). Inputs were further coded to reflect disciplinary origin and the regulatory decision or benefit-risk determination (i.e., output) that they linked to. For the process activities identified for each review issue, the analysis captured if they were purposive (i.e., preplanned or contained built-in process/workflow steps) or not.

Following this initial coding, interview participants were contacted to complete the informed consent process and schedule a semi-structured interview [28]. Interviews took between 30 and 60 min and involved six review team members for the RINVOQ case and seven review team members for the TAUVID case. A set of semi-structured interview questions were utilized to guide the interview and ensure adequate data collection around the IPO domains (see Supplementary Materials). Audio recordings from the interviews were transcribed, analyzed, and coded for review issues, inputs, outputs, and/or process activities.

Transcripts and review documents were reanalyzed and recoded several times to identify any additional data associated with newly identified inputs, outputs, and/or process activities [27,29]. This iterative coding, analysis, and interpretation process ensured that an accurate picture of each review issue and its integration could be collected. The final analysis step encompassed a review of codes to inventory them in a database adapted from the O’Rourke et al. framework for cross-disciplinary integration [20].

The IPO analysis and inventory were used to create visual IPO models (i.e., Input>Process>Output) of the integration for each review issue. These IPO models were distributed to interview participants to review and validate for member checking. Instructions were provided to the interview participants to identify any inaccuracies or additional feedback about the models. Responses from the member checking were received from all interview participants prior to considering the models validated. Minor edits were received from a subset of participants that only resulted in changes to some text. This member checking in addition to the document review and interviews helped to triangulate the qualitative data collected and enhance reliability and credibility of the analysis.

The data collected and analyzed using the contextualized O’Rourke et al. framework and IPO model also enabled the visual representation of review issue integration for each case using Sankey flow models, similar to an approach used by Laursen [30]. These flow models have been used to describe cross-disciplinary reasoning and offer a unique visual representation of the integration that occurs from inputs to outputs.

The colors in the Sankey diagram are arbitrarily selected to help the interpreter distinguish between the different disciplines and their contributions to the inputs, then the flow of inputs into process activities, and lastly the flow of those process activities to the final output. The width of the bars or flows is driven in part by the number of disciplinary contributions to the inputs, but mostly by the number of process activities these inputs were involved in. All disciplinary contributions and inputs were counted equally, and so the width of the input “flow” reflects mostly the degree to which this input was involved in the process activities. As Sankey diagrams were originally intended for the modeling of thermodynamic systems where energy was contained or conserved in the system, the remaining flow widths are all driven by the width of the Inputs and how these flow through the rest of the integration model [31].

This study was evaluated and considered exempt by the FDA IRB and GWU IRB. It is important to note that the lead researcher in this study was the lead for the FDA New Drugs Regulatory Program Modernization, which to date has implemented a structural reorganization of the Office of New Drugs, the lead FDA office for the review of new drug products. Given this close connection to the development of the interdisciplinary review, which was by design intended to create more cross-disciplinary integration, the research potentially has implicit biases that required several mitigations to minimize. Specifically, the mitigations to decrease bias and strengthen the validity of this study were the random selection of cases for the study, member checking to validate data collection, an interrater during initial coding, the use of multiple methods to triangulate data, and the inclusion of the researcher’s subjectivity in the informed consent process and communications with the participants [32].

## Results

The two cases selected are described in Table 1. While the products, indications, responsible review organization, and other application-specific administrative data on these cases are different, the review teams are quite similar.

**Table 1.** Application cases for comparative case study

Case application	NDA 211675	NDA 212123
Product	*RINVOQ* (upadacitinib), a small molecule inhibitor of the Janus-associated kinases	*TAUVID* (flortaucipir F-18), a benzimidazole pyrimidine derivative small molecule labeled with fluorine-18 for imaging
Indication	For the treatment of adult patients with moderately to severely active rheumatoid arthritis	For use with positron emission tomography imaging of the brain to estimate the density and distribution of aggregated tau neurofibril tangles in adult patients with cognitive impairment who are being evaluated for Alzheimer’s disease
Review type	Multidisciplinary review	Interdisciplinary review
Submit date	December 19, 2018	September 30, 2019
Approval date	August 16, 2019	May 28, 2020
Review timeline	8-month priority review for new molecular entity	8-month priority review for new molecular entity
Sponsor	AbbVie, Inc	Avid Pharmaceuticals, Inc
Division	Division of Pulmonary, Allergy, and Rheumatology Products	Division of Medical Imaging and Radiation Medicine
Review team	cross-disciplinary team leader regulatory project manager clinical reviewer statistical reviewer clinical pharmacology reviewer two pharmacometrics reviewers pharmacogenomics reviewer nonclinical pharmacology/toxicology reviewer drug substance reviewer drug product reviewer process/microbiology/facility reviewer biopharmaceutics reviewer	cross-disciplinary team leader regulatory project manager clinical reviewer statistical reviewer clinical pharmacology reviewer nonclinical pharmacology/toxicology reviewer drug substance reviewer drug product reviewer process/microbiology/facility reviewer

NDA, new drug application.

Six cross-disciplinary review issues were identified in the RINVOQ case based on the number of times an issue was referenced across the reviews and the impact it had on the team’s assessment as conveyed in interviews. These six review issues, which were all considered resolved, were analyzed as involving a complete input to output process. In contrast to the multiple RINVOQ application review documents, only one review document, the integrated review, is generated by the review team conducting an interdisciplinary assessment. In addition, the integrated review document is structured to be much more focused on documenting the review issues identified over the course of the review. As a result, the review issues of the integrated review were more easily identified from the initial document analysis. Six review issues were identified in the TAUVID case and like RINVOQ all were considered resolved and could be modeled as a complete input to output process (see Table 2).

**Table 2.** RINVOQ and TAUVID review issues

RINVOQ review issue #	Description
1	*Embryofetal issue:* a signal of teratogenicity, or risk to fetus, was identified and considered more significant than other products in the class
2	*30 vs 15 mg Issue:* studies were conducted for two doses of the product and the benefits/risks were assessed for both
3	*Formulation bridging issue:* a formulation change occurred during development and studies were conducted with a different formulation than the to-be-marketed formulation, which required an assessment for equivalence
4	*CYP3A4 issue:* studies indicated that coadministration of CYP3A4 inhibitors or inducers could have an impact on the pharmacokinetics of the new drug product
5	*Impurities issue:* numerous impurities were noted in the finished drug product and required comprehensive toxicologic assessment
6	*JAK class safety issue:* the Janus-associated kinases (JAK) inhibitors class of drug products were known to be associated with toxicities and these toxicities needed to be carefully assessed in the proposed product
**TAUVID review issue #**	**Description**
1	*User guide for positron emission topography (PET) image display issue:* issue related to the accuracy and usability of the sponsor-submitted user guide for the interpretation of medical imaging results following use of the drug
2	*Efficacy evidence issue 1:* this was an issue related to the evidence submitted to support a proposed claim or intended use of the product
3	*Efficacy evidence issue 2:* this was an issue related to the evidence submitted to support a proposed claim or intended use of the product
4	*CTE misdiagnosis Issue:* review team members identified a potential safety issue related to the potential to misdiagnose chronic traumatic encephalopathy (CTE) with the product
5	*Effect of monoamine oxidase (MAO) inhibitors issue:* due to known characteristics of other MAO inhibitors, TAUVID off-target binding was assessed to characterize specific inhibition with TAUVID
6	*QT interval prolongation issue:* the applicant reported small but statistically significant increase in events of QT interval prolongation that needed to be evaluated as a potential risk to patients

Six review issues were identified from the document analysis and semi-structured interviews for both the RINVOQ and TAUVID cases. These review issues related to the overall benefit-risk assessment and either had an impact on the final benefit-risk decision for RINVOQ or TAUVID approval or could have if left unresolved.

By cataloging the inputs, process activities, and outputs of each review issue (see Table 3), the review issues were further analyzed and subsequently modeled as input/output processes for member checking. The most cross-disciplinary review issue for RINVOQ was Review Issue 2 and related to an assessment of benefits and risks of two doses for the product. It involved three inputs, three process activities, and a singular output (see Table 3). The output of the integrative team process for this review issue was the finding that the 15 mg dose had a favorable benefit-risk profile, but 30 mg dose did not.

**Table 3.** RINVOQ 30 vs 15 mg review issue input/output and process activities

Input #	Discipline	Input description	
I1	Clinical pharmacology	Exposure-response analysis	
I2	Clinical, clinical pharmacology, and statistics	Integrated safety analyses	
I3	Clinical, clinical pharmacology, and statistics	Results from five phase 3 studies	

**Process activity #**	**Process description (inputs involved)**	**Purposive**	**Integrative nature**
P1	Analysis/assessment of safety at both 15 and 30 mg independently (I1, I2, I3)	Yes	Combinatorial
P2	Analysis/assessment of benefit at both 15 and 30 mg independently (I1, I3)	Yes	Combinatorial
P3	Comparative analysis of adverse event profile between 15 and 30 mg doses (I1, I2, I3)	Yes	Combinatorial
**Output #**	**Description**		
O1	FDA approval and labeling for the 15 mg dose		

The most cross-disciplinary review issue for TAUVID was Review Issue 2, an assessment of evidence of efficacy. This review issue included four inputs from four disciplines, three process activities, and two outputs (see Table 4). The outputs of the integrative team process for this review issue were FDA’s decision on the approvability of the proposed indication and their revision of Prescribing Information (i.e., drug product labeling) to reflect updated instructions for image interpretation.

**Table 4.** TAUVID efficacy evidence review issue 2 input/output and process activities

Input #	Discipline	Input description	
I1	Clinical, CDRH, and statistics	Two phase 3 studies (A05C and PX01), including study reports, case report forms, and line item data	
I2	Statistics	Sensitivity analyses	
I3	Regulatory, clinical, and statistics	Presubmission meeting discussions	
I4	Clinical and statistics	Additional data and information requested by the review team	

**Process activity #**	**Process description (inputs involved)**	**Purposive**	**Integrative nature**
P1	Multiple review team discussions primarily between clinical and statistics, led by clinical (I1, I2, I3)	Yes	Synthetic
P2	CDRH was consulted to assess the devices used in the clinical studies (I1)	Yes	Combinatorial
P3	Review team held negotiations with the Applicant (I1, I3, I4)	Yes	Analytic
**Output #**	**Description**		
O1	FDA recommendation to applicant		
O2	FDA decision and labeling to reflect findings		

CDRH, Center for Devices and Radiological Health.

The Sankey diagrams of these two review issues include a more visual representation of the links or flows between inputs, process activities, and the output(s).

As can be seen in the Sankey figure, two of the RINVOQ process activities (P1 and P3; see Table 3) were inclusive of all inputs. In the RINVOQ review issue, three inputs related to the two doses were processed via additional analyses or assessments for safety risks, effectiveness benefits, and then both individually and comparatively for benefit-risk assessments. It is a routine practice for disciplines to conduct their analyses/assessments independently in this way before bringing their findings together to make a consensus decision. Because there is little change to the inputs during the process activities, the process activities were considered combinatorial regarding their integrative nature. Also, interestingly, in the Sankey diagram for the RINVOQ review issue is the contribution of the clinical pharmacology discipline to all inputs and subsequent incorporation in all process activities. This clearly indicates extensive reliance on this discipline in the overall review issue.

The Sankey modeling of the integration for the TAUVID review issue illustrates an even more visually dynamic picture of activity. Also of note, this review issue involved one process activity that included all inputs like the two process activities for the RINVOQ review issue; however, the TAUVID review issue involves a larger number of inputs. It is worth mentioning that process activity 3 (P3) for the TAUVID review issue was analytic, and so disintegrative; as discussed earlier, analytic changes aim to break an input down into its constituent parts or to differentiate between inputs, and if the Sankey diagrams could reflect this sort of disintegration of inputs then the model might look quite different (i.e., a coming apart of inputs into subinputs or something to this effect). As it is, Sankey diagrams aim to conserve all inputs in the input, process, and output model as if they were energy in a closed system, given the roots of Sankey diagraming in thermodynamics [31].

The RINVOQ integration surrounding the six identified cross-disciplinary review issues was mostly cross-disciplinary from the outset, meaning more than one discipline was involved in each review issue. Two review issues, review issue 4 and 6, involved unidisciplinary inputs, but over the course of the review became cross-disciplinary as additional team members provided input on the assessment. In contrast, the integration surrounding the six identified cross-disciplinary review issues in the TAUVID case were entirely cross-disciplinary from the outset. In further comparing inputs, the TAUVID interdisciplinary case involved a larger number of inputs than the RINVOQ multidisciplinary case, but the mean and median number of inputs per issue were similar (see Table 5). We suspect that the use of the interdisciplinary approach to assessing applications led to a greater sensitivity to cross-disciplinary issues, resulting in the larger number of input types.

**Table 5.** Comparison of inputs and process activities

Variable	RINVOQ	TAUVID
Number of inputs	17	20
Number of inputs per issue	Mean: 3 Median: 3	Mean: 3 Median: 4
Number of disciplines contributing	Mean: 4 Median: 2	Mean: 4 Median: 4
Degree of discipline involvement by input contributions	Clin Pharm (9) Clinical (4) Pharm/Tox (4) Statistics (3) Biopharm (3) Chemistry (1)	Clinical (14) Statistics (8) CDRH (5) Clin Pharm (4) Pharm/Tox (4) Regulatory (2) DMEPA (1) Policy (1) QT IRT (1)
Number of process activities per case	13	16
Number of process activities per issue	Mean: 2 Median: 2	Mean: 3 Median: 3
Multi-input process activities	7 (54%)	14 (88%)
Cross-disciplinary process activities	8 (62%)	16 (100%)
Purposive process activities	8 (62%)	11 (69%)
Combinatorial process activities	6 (46%)	5 (31%)
Synthetic process activities	4 (31%)	8 (50%)
Analytic process activities	1 (8%)	3 (19%)
Process activities with no change	2*	0

* Represents 13% of the process activities and were not assessable for integrative nature [20].

Variables listed include those noted in O’Rourke et al. for the qualitative and quantitative analysis of integration in the input-process-output model, such as count of process activities, purposiveness, integrative, or disintegrative nature (i.e., synthetic, combinatorial, analytic).

CDRH, Center for Devices and Radiological Health; DMEPA, Division of Medication Error Prevention and Analysis; QT IRT, QT Interdisciplinary Review Team.

In addition, the median number of disciplines that contributed to inputs in the instances of integration for the TAUVID interdisciplinary case was double that of the RINVOQ multidisciplinary case. This difference, coupled with input from interviews related to TAUVID process activities, seems to indicate that disciplinary expertise was brought to bear on review issues through more deliberate collaboration (e.g., review team discussions, collaborative writing). That the RINVOQ multidisciplinary case included more than one instance of integration around a review issue that began as unidisciplinary is also an interesting difference. This finding may reflect a key temporal difference in the review process between the two cases, where earlier interdisciplinary interactions in the interdisciplinary review case render unnecessary late review cycle collaboration and integration.

In the interdisciplinary review, by design, the review teams conduct several new integrated activities: a benefit-risk scoping meeting prior to the filing of the NDA (i.e., determination that the application is on its face complete) or original BLA, and then for each identified review issue, a JAM is held, which focuses on the issue and includes all relevant team members [9]. The benefit-risk scoping meeting is an opportunity for the review team to discuss their initial view of the application and potential review issues (i.e., “problems”). Subsequent JAM meetings are used to discuss the scoped-out review issues in a focused fashion.

As can be seen in Table 5, the TAUVID interdisciplinary case involved a greater number of process activities in total and per review issue. Given the additional integrated activities mentioned above for the new interdisciplinary review, this was an expected finding. There was a dramatic increase in the number of process activities that involved multiple inputs and multiple disciplines, which suggests an increase in collaboration in the interdisciplinary review. There was also a much larger number of synthetic process activities – double, in fact – in the TAUVID interdisciplinary case than in the RINVOQ multidisciplinary case, suggesting a higher degree of integration.

## Discussion

**Fig. 5.**
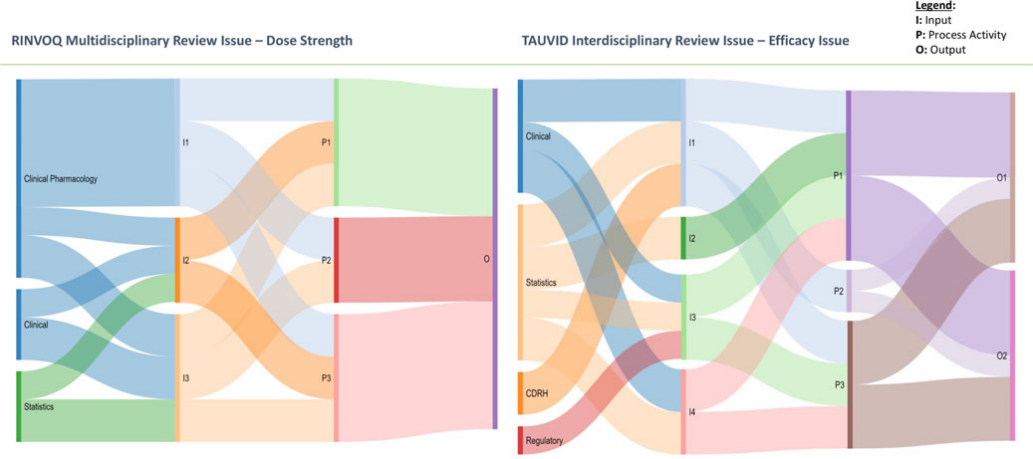
Side-by-side integration comparison. More inputs were brought to bear with TAUVID in a greater number of outputs produced but over the same priority review period of 8 months, suggesting a more “active” or involved integration process. CDRH, Center for Devices and Radiological Health.

This study sought to identify and characterize integration in two approaches to the FDA’s assessment of marketing applications for new drug products, where improved integration was the main reason for moving from the traditional multidisciplinary approach to the new interdisciplinary approach. It applied the IPO framework and model to successfully identify and characterize twelve instances of integration in two cases of new drug product application reviews, the RINVOQ multidisciplinary review, and the TAUVID interdisciplinary review. This finding confirms that integration is occurring in FDA new drug product reviews around benefit-risk review issues, as expected. The study also demonstrated observable differences in integration between a multidisciplinary case and an interdisciplinary case of FDA marketing application reviews. These differences may be a result of intentional changes to collaborative process activities and documentation in the new interdisciplinary review for marketing applications. This can be visually observed by placing the “most integrative” and most cross-disciplinary review issue from the RINVOQ case side-by-side with the most integrative and cross-disciplinary TAUVID review issue (see Fig. 5). The process for the TAUVID review issue appears visibly more active in the Sankey diagram, with more connections and more crossing flows, reflecting greater cross-disciplinary collaboration in the process activities. In addition, with more inputs and process activities but the same overall timeframe for the overall review issue’s resolution, there appears to be more integrative activity in the TAUVID review issue, as expected.

Furthermore, this study’s focus on collaborative, cross-disciplinary integration in cases of FDA’s multidisciplinary and interdisciplinary reviews of new drug product marketing applications indicates some promise for using the O’Rourke et al. framework and associated IPO model as an approach to evaluating cross-disciplinary integration in future FDA-related collaborative research efforts. This case study offered an opportunity to test the O’Rourke model to characterize integration in a situation where integration was expected to be different due to the implementation of more interdisciplinary interventions in a team’s process. We learned that it could do this and that with the right adaptation (i.e., coupling with the BRF), it could be used to illuminate FDA reviews of new drug products.

These findings are important information for FDA’s ongoing phased implementation of the interdisciplinary review approach. As the FDA continues to implement and refine the interdisciplinary process and integrated review template for its assessment of marketing applications, it can rely on the cross-disciplinary integration framework and IPO model to objectively characterize integration, a desired outcome, in the completed new drug product reviews. This evaluative approach will help the FDA weigh any tradeoffs of the new interdisciplinary approach. Particularly, by using the IPO model and Sankey diagram to visualize integration, FDA and its stakeholders have a new tool to visualize the integration that occurred relative to benefit and risk review issues that impacted the FDA’s regulatory decision-making.

For those researchers interested in evaluating the impact of team science interventions targeting the promotion of integration, this study suggests the value of applying the O’Rourke et al. IPO model to identify and characterize cross-disciplinary integration. We expected the TAUVID review to be more integrative than the RINVOQ review, and the IPO model characterized differences in integration that aligned with this expectation. Applying this model in additional contexts will help further assess its value as a way of identifying and characterizing cross-disciplinary integration. To answer the increasingly loud call for cross-disciplinary research to address complex, global challenges, we must identify a way to track and evaluate the integration that will be at heart of it.

## Conclusion

The O’Rourke et al. framework and modeling approach for evaluating integration, due to its generality, lends itself to broad applicability in multiple contexts as suggested by this study. The framework and IPO model identify multiple instances of integration in both a multidisciplinary case and interdisciplinary case of FDA cross-disciplinary team science. And, it has been found that the process and collaborative documentation of the interdisciplinary assessment for marketing applications has led to observable differences in integration in these cases.

Science-of-Team-Science seeks to further understand the circumstances associated with the effectiveness of collaborative team science and manage those circumstances to promote improved outcomes for teams [33]. This study has addressed both goals by practically applying the O’Rourke et al. philosophical framework for cross-disciplinary integration. As another important highlight, in scientific endeavors the provenance of new evidence is almost as important as the evidence itself. With the increasing reliance on teams to achieve interdisciplinary and transdisciplinary solutions to complex problems, it is important to identify and describe the processes that lead to integrative outputs of collaborative research, since this demonstrates that we can understand the integration that occurred and that we are in control of it; otherwise, it is just fortuitous that it occurs.

## Supplementary material

For supplementary material accompanying this paper visit https://doi.org/10.1017/cts.2021.861. click here to view supplementary material
