# Supplementary material for: Evaluating integration in collaborative cross-disciplinary FDA new drug reviews using an input-process-output model
Source: J Clin Transl Sci. 2021 Sep 30;5(1):e199. doi: 10.1017/cts.2021.861 (PMC8727715; doi:10.1017/cts.2021.861)
Supplement: Supplementary file 1 [file S205986612100861Xsup001.pptx]

## Slide 1
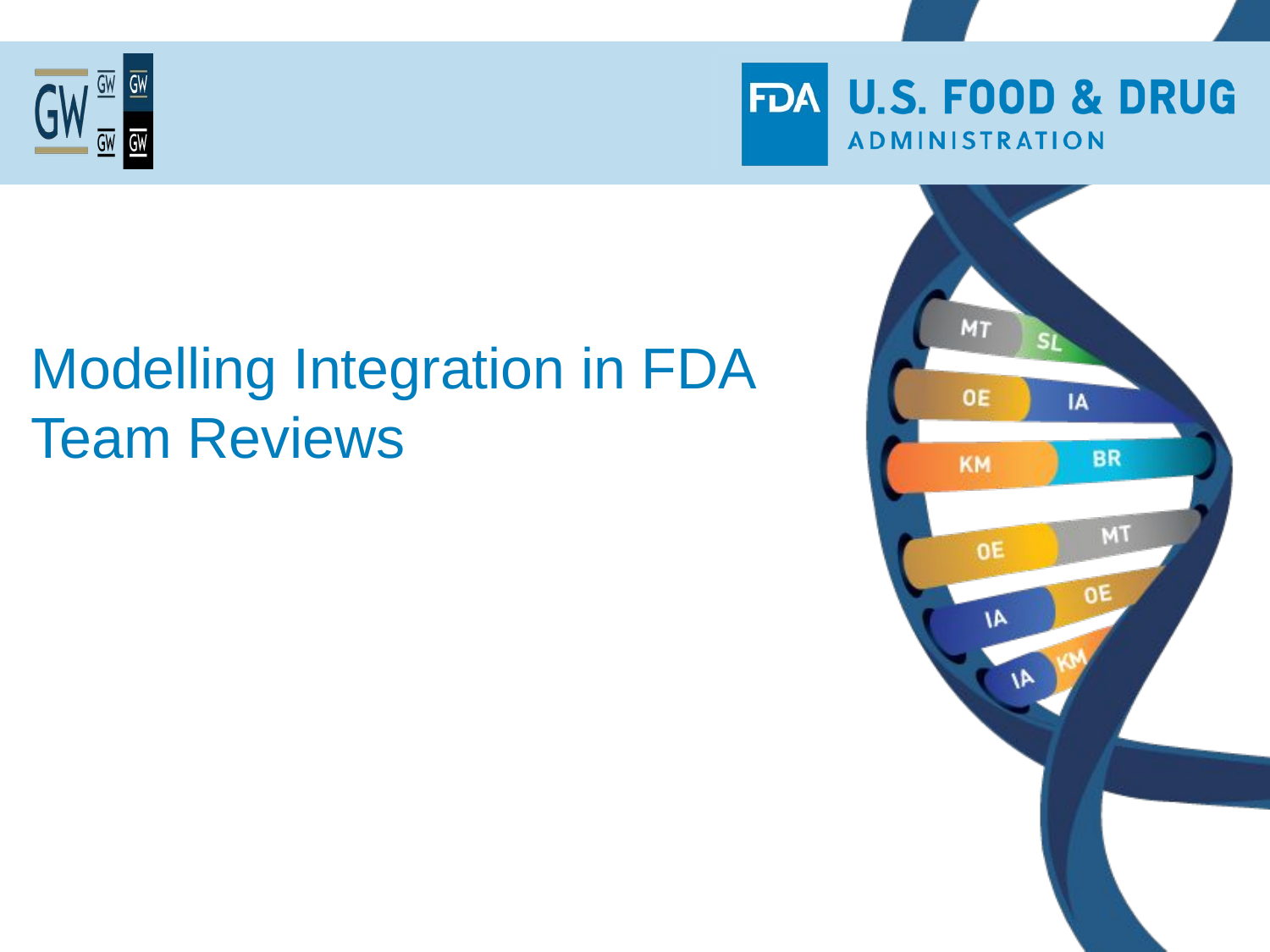

# Modelling Integration in FDA Team Reviews

## Slide 2
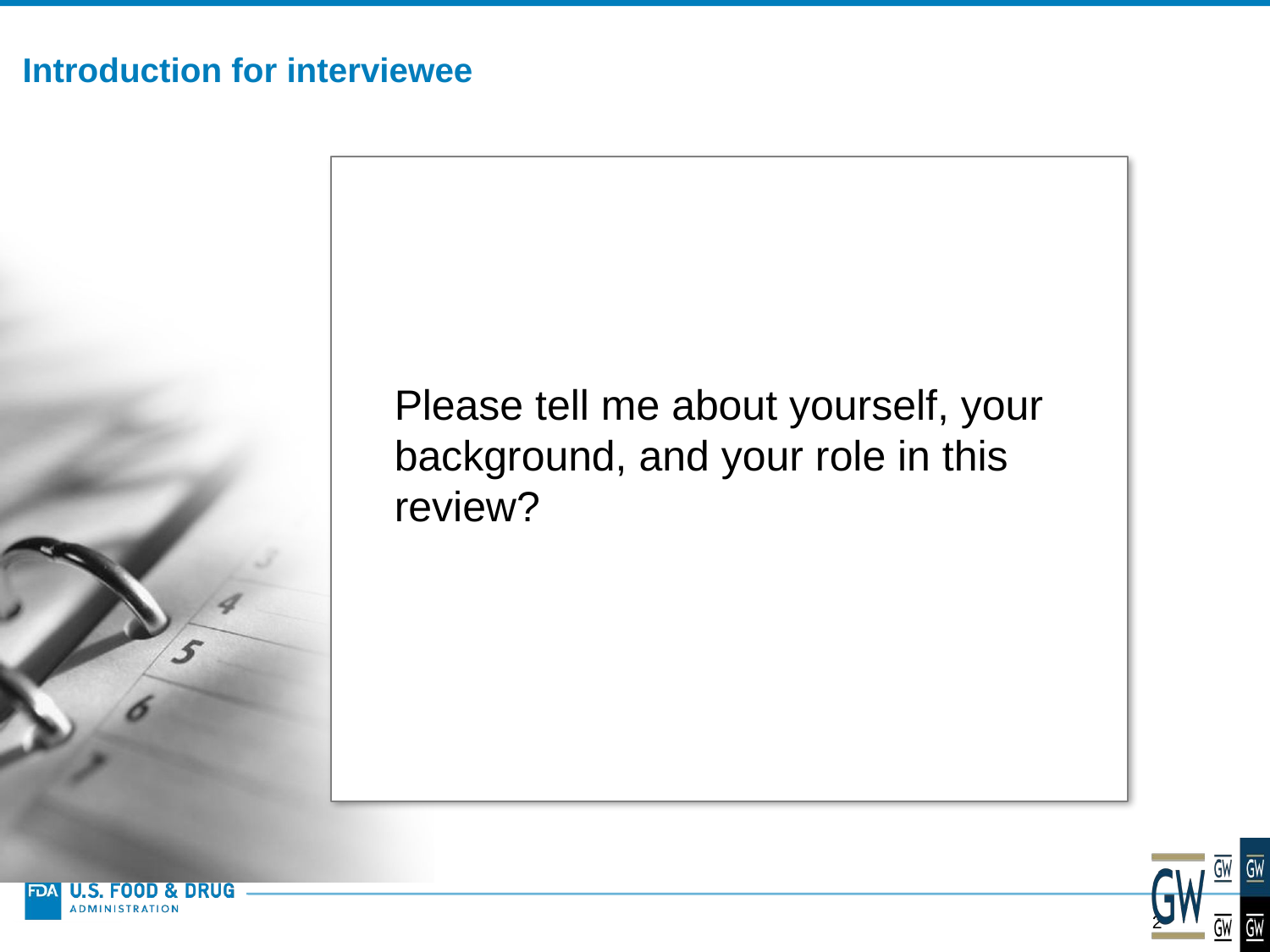

# Introduction for interviewee
Please tell me about yourself, your background, and your role in this review?

## Slide 3
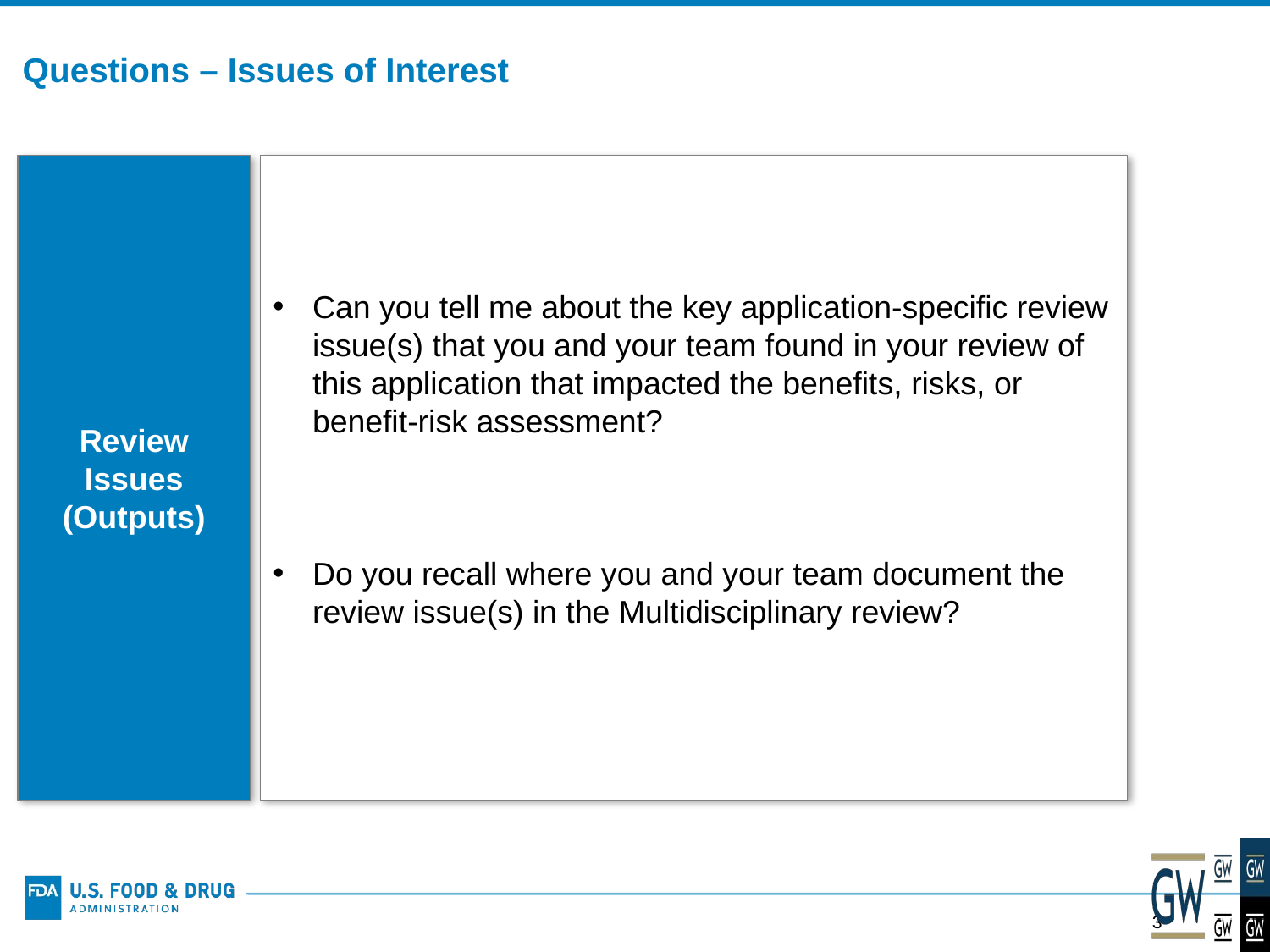

# Questions – Issues of Interest
Review Issues (Outputs)
Can you tell me about the key application-specific review issue(s) that you and your team found in your review of this application that impacted the benefits, risks, or benefit-risk assessment?
Do you recall where you and your team document the review issue(s) in the Multidisciplinary review?

## Slide 4
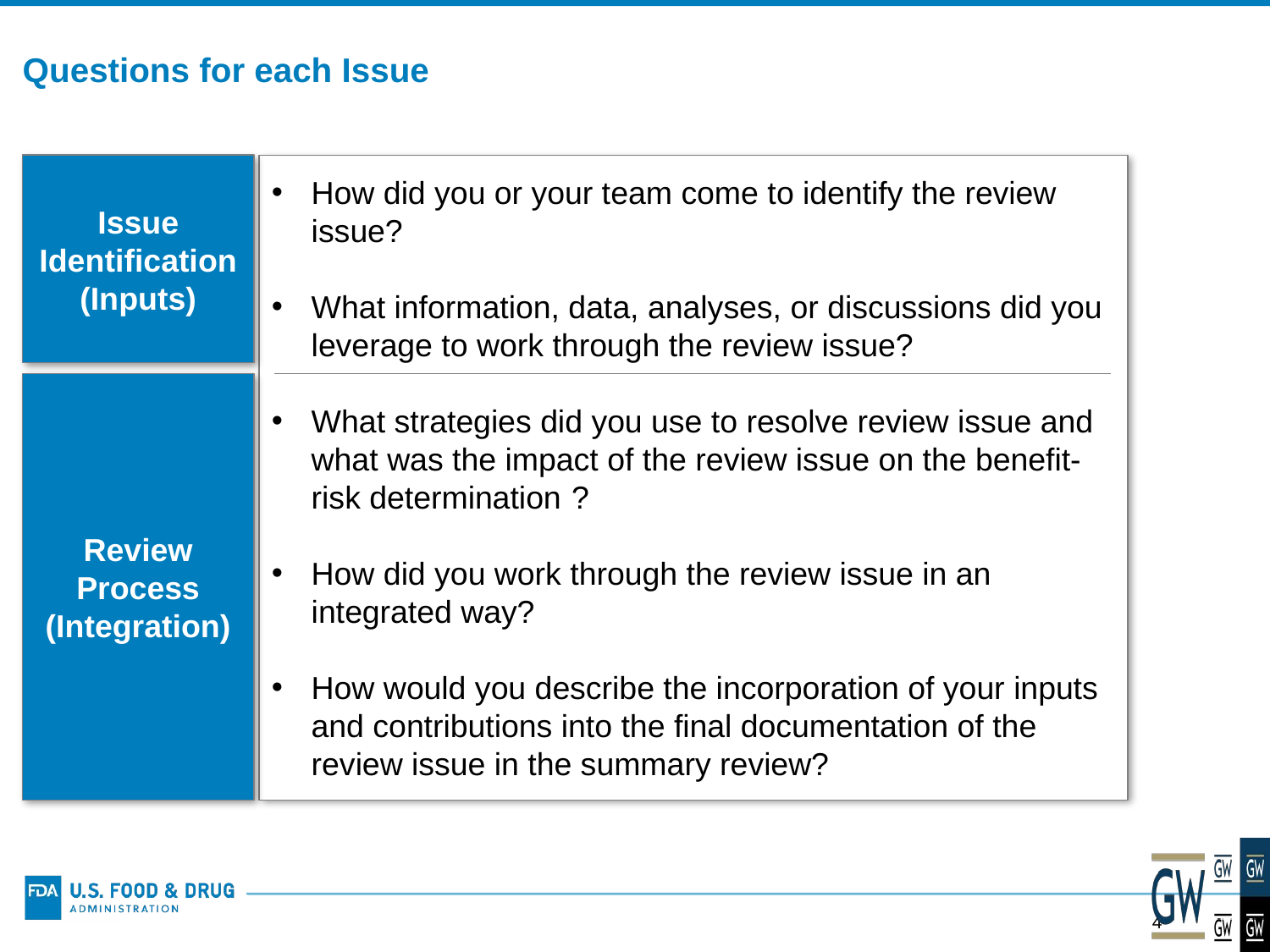

# Questions for each Issue
Issue
Identification
(Inputs)
How did you or your team come to identify the review issue?
What information, data, analyses, or discussions did you leverage to work through the review issue?
What strategies did you use to resolve review issue and what was the impact of the review issue on the benefit-risk determination  ?
How did you work through the review issue in an integrated way?
How would you describe the incorporation of your inputs and contributions into the final documentation of the review issue in the summary review?
Review Process (Integration)

## Slide 5
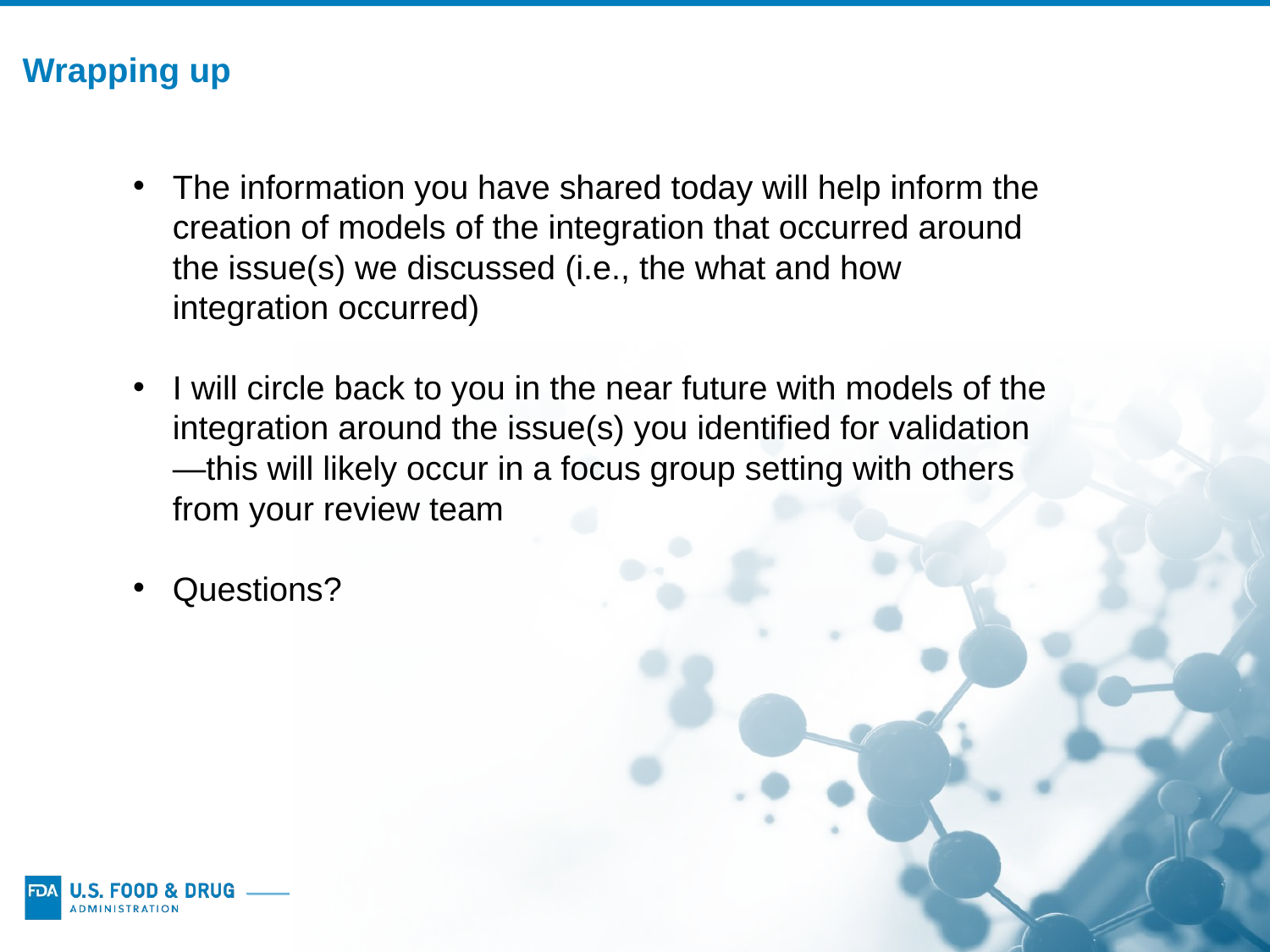

# Wrapping up
The information you have shared today will help inform the creation of models of the integration that occurred around the issue(s) we discussed (i.e., the what and how integration occurred)
I will circle back to you in the near future with models of the integration around the issue(s) you identified for validation—this will likely occur in a focus group setting with others from your review team
Questions?
